# The Sero-epidemiology of *Coxiella burnetii* in Humans and Cattle, Western Kenya: Evidence from a Cross-Sectional Study

**DOI:** 10.1371/journal.pntd.0005032

**Published:** 2016-10-07

**Authors:** Nicola A. Wardrop, Lian F. Thomas, Elizabeth A. J. Cook, William A. de Glanville, Peter M. Atkinson, Claire N. Wamae, Eric M. Fèvre

**Affiliations:** 1 Geography and Environment, University of Southampton, Southampton, United Kingdom; 2 Centre for Immunity, Infection and Evolution, University of Edinburgh, Edinburgh, United Kingdom; 3 International Livestock Research Institute, Nairobi, Kenya; 4 Institute of Biodiversity, Animal Health and Comparative Medicine, University of Glasgow, Glasgow, United Kingdom; 5 Faculty of Science and Technology, Lancaster University, Lancaster, United Kingdom; 6 School of Geography, Archaeology and Palaeoecology, Queen's University Belfast, Belfast, United Kingdom; 7 Centre for Microbiology Research, Kenya Medical Research Institute, Nairobi, Kenya; 8 Mount Kenya University, Thika, Kenya; 9 Institute of Infection and Global Health, University of Liverpool, Liverpool, United Kingdom; University of California San Diego School of Medicine, UNITED STATES

## Abstract

Evidence suggests that the intracellular bacterial pathogen *Coxiella burnetii* (which causes Q fever) is widespread, with a near global distribution. While there has been increasing attention to Q fever epidemiology in high-income settings, a recent systematic review highlighted significant gaps in our understanding of the prevalence, spatial distribution and risk factors for Q fever infection across Africa. This research aimed to provide a One Health assessment of Q fever epidemiology in parts of Western and Nyanza Provinces, Western Kenya, in cattle and humans. A cross-sectional survey was conducted: serum samples from 2049 humans and 955 cattle in 416 homesteads were analysed for *C*. *burnetii* antibodies. Questionnaires covering demographic, socio-economic and husbandry information were also administered. These data were linked to environmental datasets based on geographical locations (e.g., land cover). Correlation and spatial-cross correlation analyses were applied to assess the potential link between cattle and human seroprevalence. Multilevel regression analysis was used to assess the relationships between a range of socio-economic, demographic and environmental factors and sero-positivity in both humans and animals. The overall sero-prevalence of *C*. *burnetii* was 2.5% in humans and 10.5% in cattle, but we found no evidence of correlation between cattle and human seroprevalence either within households, or when incorporating spatial proximity to other households in the survey. Multilevel modelling indicated the importance of several factors for exposure to the organism. Cattle obtained from market (as opposed to those bred in their homestead) and those residing in areas with lower precipitation levels had the highest sero-prevalence. For humans, the youngest age group had the highest odds of seropositivity, variations were observed between ethnic groups, and frequent livestock contact (specifically grazing and dealing with abortion material) was also a risk factor. These results illustrate endemicity of *C*. *burnetii* in western Kenya, although prevalence is relatively low. The analysis indicates that while environmental factors may play a role in cattle exposure patterns, human exposure patterns are likely to be driven more strongly by livestock contacts. The implication of livestock markets in cattle exposure risks suggests these may be a suitable target for interventions.

## Author Summary

The bacteria *Coxiella burnetii* has a widespread distribution and causes illness in both humans and livestock (Q Fever), including long-term effects in a proportion of cases. Despite a recent resurgence in interest in a European context, there is a significant lack of understanding of the prevalence of exposure, burden of disease, or epidemiological risk factors in low-income settings. Our study provides much needed new evidence, reporting seroprevalence in a linked human and cattle population in western Kenya and identifying factors associated with increased seroprevalence in humans and cattle within this setting. Our results indicate that environmental factors may play a role in patterns of exposure in cattle populations in western Kenya, where cattle in areas with less rainfall were more likely to have evidence of previous exposure to the bacteria. However, human exposure is more likely to be influenced by livestock contact patterns. In addition, cattle brought onto a homestead following purchase at a market or another homestead had higher seroprevalence than those bred on the homestead. Further research on the role of livestock markets in disease spread is required and may form the basis for the future development of Q Fever control measures.

## Introduction

*Coxiella burnetii*, the etiological agent of ‘Q fever’, has caused several large scale outbreaks within Europe over recent years [1] and contributes an ongoing human and livestock health burden in many regions [2]. The distribution of *C*. *burnetii* is thought to be global, with the exception of Antarctica and New Zealand [1,3]. The pathogen is zoonotic and its main reservoir, and source of infection for humans, exists in livestock populations, although a wide range of other wild and domestic animals, birds, amphibians and arthropods can carry the bacterium [4]. Despite its ubiquitous nature, significant gaps in our understanding of the epidemiology of this pathogen still remain, particularly in resource-poor settings [5].

Infection in livestock animals is predominantly asymptomatic, but can result in reproductive disorders, such as spontaneous abortion, weak offspring or infertility [6]. In humans, up to 40% of those infected will develop acute Q fever, which manifests as a non-specific febrile illness, pneumonia and/or hepatitis [7,8]. Acute Q fever is normally self-limiting, but 2–5% of cases can experience more severe symptoms [7]. Furthermore, approximately 2% of patients will develop persistent focalized infections, including mainly endocarditis and vascular infection. A post-infection fatigue syndrome has been reported without any evidence for persisting infection in this context [7,9–11]. The clinical picture of Q fever varies geographically, and also depends on host factors, such as immune status and the presence of pre-existing conditions [12].

Infected animals excrete *C*. *burnetii* in their milk, urine and faeces. In addition, particularly high numbers of the organism are found in the birthing materials (e.g. placenta) of pregnant animals. The non-replicating, small-cell variant of *C*. *burnetii*, which is found outside of host animals, is extremely resistant to environmental conditions and can remain infective for several months. Thus, a contaminated environment can act as a long-term reservoir [8,13]. Onward transmission normally occurs via the respiratory route due to the aerosolisation of contaminated materials, including dust, and as few as 1–10 organisms are required for infection. Previous studies have also indicated the dispersal of *C*. *burnetii* over several kilometres by wind [14,15]. Transmission due to the consumption of unpasteurised dairy products, or via the bite of an infected tick, is also possible.

A recent systematic review of *C*. *burnetii* epidemiology across Africa by Vanderburg *et al* [5] highlighted evidence of endemicity in cattle, small ruminants and humans across the continent, with seroprevalence ranging from 7% to 33% in sheep, 4% to 55% in cattle, and 1% to 32% in humans. Variations in seroprevalence have also been observed within relatively small distances [5,16], but few studies have examined risk factors for exposure in human and livestock populations simultaneously and there is a poor understanding of the reasons behind the observed heterogeneity in seroprevalence [5]. Risk factors for seropositivity in cattle which have been identified (specific to the continent of Africa) include: older age groups [17,18]; female gender; presence of buffalo near grazing areas; and ethnicity of livestock owner [17]. Identified human risk factors for exposure to *C*. *burnetii* in African settings include: illiteracy of mothers (risk factor for children) [19]; male gender; camel breeding [20]; and younger age group [16,20]. There has been greater attention to *C*. *burnetii* epidemiology in high-income settings (e.g. Europe, Australia), but it is likely that major epidemiological differences will exist between different settings for a variety of reasons, including husbandry practices and patterns of human and livestock density.

A recent study in Rarienda district, western Kenya detected a seroprevalence of 28.3% in cattle, 32% in goats, 18.2% in sheep and 30.9% in humans, with seroprevalence increasing with age for livestock [21]. The same study also detected *C*. *burnetii* DNA in ticks collected from cattle and dogs. Another study in Bungoma, western Kenya conducted between 2011 and 2012 identified a seroprevalence of 12.9% amongst a sample of febrile children between one and 12 years of age and evidence of acute Q Fever in 8.9% of this sample [22], indicating that Q Fever may be an important cause of non-malarial febrile illness in this area. In addition, a survey carried out in Laikipia county in 2011 detected seroprevalences ranging from 0% to 4% in cattle; 13% to 20% in sheep; 31% to 40% in goats; and 5% to 46% in camels [23]. A recent review has highlighted the sparse availability of high quality epidemiological data, despite evidence that exposure to *C*. *burnetii* is common in both human and livestock populations within Kenya [24]. Based on existing gaps in understanding and a lack of recent data, this paper aimed to (a) assess the seroprevalence of *C*. *burnetii* in linked human and cattle populations in a rural area of western Kenya and (b) determine the socio-economic, behavioural and environmental factors correlated with exposure in humans and cattle. By using a linked human-livestock survey, this study takes a One Health approach, enabling examination of household level linkages between cattle and human exposure, and the detailed examination of risk factors in both livestock and humans.

## Materials and Methods

### Data

**Fig 1:**
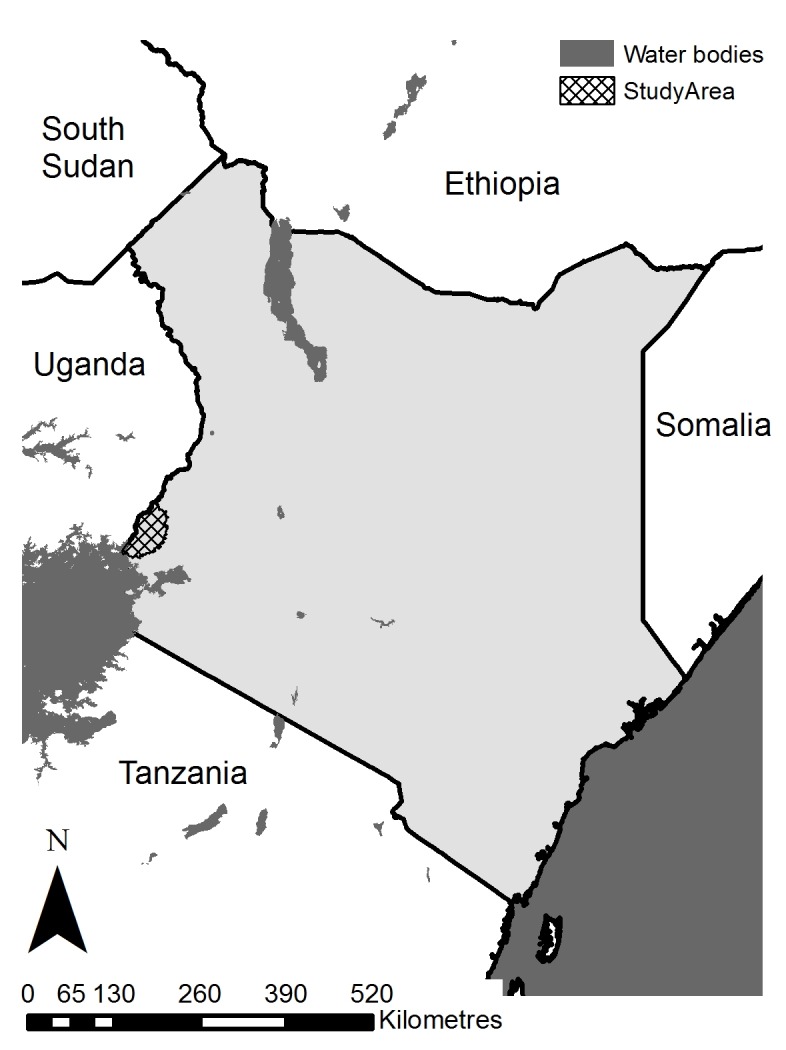
Map of Kenya indicating the study area (hatched area).

This One Health study was conducted in parts of Western and Nyanza provinces in western Kenya (following restructuring of administrative areas in Kenya, the area covered includes parts of Busia, Siaya and Bungoma counties; see Fig 1) with a population density of approximately 500 per km^2^. Subsistence agriculture (mixed crop-livestock) is the predominant occupation within the study population, and several ethnic groups are present, mainly Luhya, Luo, Teso and Samia. The area experiences a bimodal climate: rainy seasons run from March to May and August to November, and the average temperature is approximately 22°C (range 14°C to 30°C) [25]. This area of Kenya was selected as representative of the Lake Victoria basin ecosystem, with high population density and a predominantly crop-livestock production system. For logistical reasons, the final study area covered a radius of 45 km (on the Kenyan side of the border) from the project laboratory in Busia town. The study area borders Uganda, and, given the porous nature of the Kenyan-Ugandan border, transfer of livestock across the border is likely.

A cross-sectional serological survey, with a clustered sampling design, was carried out in both humans and cattle. The sample size was powered to account for an expected minimum prevalence of 5% for bovine infection (the survey was designed to detect several zoonotic diseases in the study area), with a standard error of 2%. The design effect, to account for the likely increase in standard error due to clustering, was set to 3. This resulted in a required sample size of 1365 cattle, which were expected to be recruited from 412 randomly selected households, based on local herd size and frequency of cattle ownership estimates. The human sample size was incidental to this. A total of 416 households were selected randomly from the study area, with the number of households per sub-location (the smallest administrative area in Kenya) weighted by the cattle population of each sub-location (i.e. more households selected in sub-locations with more livestock). Spatial points were generated randomly in each sub-location, and the household closest to each of these points was included in the study. The spatial coordinates of the study households were recorded precisely using a handheld global positioning system. Following informed consent, all humans over 5 years of age, apart from women in the third trimester of pregnancy (determined by self-report), were included in the study, as were all cattle, excluding female cattle in the last trimester of pregnancy (determined by farmers report).

Serum samples were obtained from all participants and evidence of exposure to *C*. *burnetii* was assessed using antibody ELISA methods. Human samples were assessed using the Serion ELISA Classic *Coxiella burnetii* Phase 2 IgG kit (Virion/Serion, Würzburg, Germany). A correction factor, which was calculated by dividing the reference optical density (OD) of the standard serum with the current OD of the standard serum, was used to account for inter-assay variability. All measured values of samples were multiplied by the correction factor and subsequently used to assign samples as seropositive or seronegative, as recommended by the manufacturer. Cattle samples were assessed using the CHEKIT Q Fever Antibody ELISA Test Kit (IDEXX Laboratories, Wetherby, UK). The OD results of duplicate samples were averaged and the following equation applied to the results, prior to determining the sero-status of each sample, based on the recommendation of the manufacturer:

Value%=ODsample–ODneg/ODpos–ODnegx100%

See S1 Appendix for further details of serological testing.

Questionnaires were administered in relation to (1) household level factors (e.g. livestock keeping; answered by a single individual per household), (2) individual human level factors (e.g. demographic information) and (3) individual cattle level factors (e.g. age, sex). See Table 1 for a full list of the individual level and household level questionnaire derived covariates used.

**Table 1:** Full list of individual and household/herd level covariates used for analysis of human and cattle seropositivity, including sources. Note that household/herd level covariates apply to all humans within a single household, and all cattle owned by that same household (although not all households own cattle).

Level	Human covariates	Cattle covariates
**Individual**	Age group*	Breed*
	Gender*	Gender*
	Ethnic background*	Origin of animal*
	Educational attainment*	Cattle herded with sheep and goats*
	Occupation*	Herding practice (dry season)*
	Frequency involved in grazing livestock*	Herding practice (wet season)*
	Frequency involved in feeding livestock*	
	Involved in milking cattle*	Provides milk for household*
	Involved in animal births*	Previously calved*
	Involved in handling animal abortus*	History of abortion*
	Involved in animal slaughter*	
	Involved in dealing with animal manure*	
	Involved in animal skinning*	
	Involved in burying dead animals*	
	Animals present in building used for sleeping*	
	Drink cow’s milk*	
	Drink goat’s milk*	
**Household/herd**		Number of human inhabitants*
	Keep cattle*	Number of cattle*
	Keep sheep*	Sheep*
	Keep goats*	Goats*
	History of abortion in herd*	History of abortion in herd*
	Seropositive cattle in herd	Seropositive human in household
	Distance to water**	Distance to water**
		Inverse distance to water**
	Distance to flooding land**	Distance to flooding land**
	% land agricultural and grassland**	% land agricultural and grassland**
	% land flooding**	% land flooding**
	% land flooding agricultural and grassland**	% land flooding agricultural and grassland**
	% land swamp**	% land swamp**
	% land woodland and shrubs**	% land woodland and shrubs**
	% land vegetated**	% land vegetated**
	% land water body**	% land water body**
	Mean temperature [25]	Mean temperature [25]
	Annual precipitation [25]	Annual precipitation [25]
	Elevation [27]	Elevation [27]
	Population density [28]	Population density [28]

*Derived from questionnaire responses

**from classified satellite imagery, as described in text [26].

Additional covariate datasets were obtained to provide information on a range of external factors thought to be relevant for *C*. *burnetii* exposure, based on previously published research (see Table 1). Land cover data [26] were created by classification of remotely sensed imagery, as described in Wardrop *et al* [29]. Spatial buffers of 1 km were created around each study homestead and the percentage of each land cover type (agricultural and grassland; flooding; flooding agricultural and grassland; swamp; woodland and shrubs; water bodies; vegetated) within each buffer was calculated using ArcMap 10 (ESRI, Redlands), to represent the landscape surrounding each homestead [29]. In addition, the Euclidean distances from each homestead to the closest water body and the closest area of flooding land were calculated. Mean temperature and total annual precipitation data were obtained from the Worldclim dataset at a spatial resolution of 1 km [25]. Population density data were obtained from the WorldPop project at a spatial resolution of 100 m [28] and elevation data were obtained from the Shuttle Radar Topography Mission (SRTM) at a spatial resolution of 90 m [27]. Covariate data were linked to the survey households based on spatial coordinates.

### Ethical approval

Ethical approval was granted by the Kenya Medical Research Institute Ethical Review Board (SC1701; human sample collection), the Animal Welfare and Ethical Review Body (AWERB) at The Roslin Institute, University of Edinburgh (approval number AWA004; cattle sample collection) and the University of Southampton ethics review committee (ID 1986; secondary data analysis). Written informed consent was obtained for all study participants (in the case of child participants, this was provided by a parent or guardian on their behalf) and individuals’ names were not recorded to ensure anonymity. The UK National Centre for the Replacement, Refinement and Reduction of Animals in Research guidelines were adhered to.

### Analysis

The serological results were mapped at households to assess the spatial patterns of seropositivity in humans and cattle. Bivariate kernel density estimation was conducted to produce spatially smoothed “relative risk” of exposure in both humans and cattle, with a fixed kernel of 4 km, using the *sparr* package in the R statistical software [30]. This method involves kernel density estimation of seropositive and seronegative individuals separately, followed by calculation of the ratio between these densities to produce the “relative risk” surface. The identification of areas with a significantly elevated relative risk of disease was assessed via the calculation of asymptotic *p*-values for the relative risk surfaces.

The Pearson’s correlation coefficient, weighted by the number of human participants in each household, was calculated to assess the correlation between human and cattle seroprevalence at the household level. Spatial cross-correlograms were calculated to assess the same, accounting for correlation between households at increasing distances (i.e. not just correlation within households, but also between households). The weighted correlation and cross-correlogram analyses were carried out using the *weights* and *ncf* packages in the R statistical software, respectively.

Univariable mixed effects logistic regression models were used to assess the relationships between seropositivity in humans and cattle, and each of the covariates listed in Table 1. Mixed-effects models allow the inclusion of a household level random effect, which accounts for the household-level clustering of observations, and enables the assessment of both individual level and household level covariates. Covariates which had statistically significant (*p* < 0.05) relationships with seropositivity in the univariable analysis were considered in the multivariable analysis. Multivariable model development was carried out in a stepwise manner: covariates were added one at a time, at each stage including the covariate which resulted in the model with the lowest Akaike Information Criterion (AIC) value and a significant improvement in model fit (*p* < 0.05, based on ANOVA model comparison). This was carried out for individual level covariates first, then household level covariates. Where covariates were correlated with one another, covariate selection was performed based on understanding of the transmission cycle and comparison of AIC values. A receiver operating characteristic (ROC) curve was created, and the area under the ROC curve (AUC) was calculated for the two final multivariable models, to provide an assessment of model fit. The regression and ROC analysis were carried out in the R statistical software using packages *lme4* and *pROC*. See S1 Checklist for a STROBE checklist.

## Results

Within the study population, cattle were kept by 55.3% of households, and the average number of cattle per household was 4.9 (excluding non-cattle keeping households; range 1 to 67). In terms of other Q-fever susceptible species, sheep & goats were present in 35.6% of households, with average herd sizes of 3.2 goats and 3.1 sheep. Overall, 66.8% of households kept livestock, increasing to 87.3% when including poultry. The average number of humans per household included in the survey was 5.08 (range 1 to 21), and only a single person was included in the survey in 7.7% of households.

### Human seropositivity

Serum samples were obtained from 2049 humans and serological evidence of exposure to *C*. *burnetii* was detected in 52, giving an overall (raw) human seroprevalence of 2.5% (95% CI = 1.9%–3.3%). The observed seroprevalence was higher in males (3.3%) than females (1.9%; Pearson's Chi-squared test *p* = 0.07, see Table 2), and was highest in the youngest age group (4.0% [5 to 14 years], 2.2% [15 to 24 years] and 1.2% [25 years plus], Pearson's Chi-squared test *p* = 0.001, see Table 2).

**Table 2:** Human serological results, by gender and age group.

Variable	Category	Seronegative	Seropositive *(seroprevalence [95% CI])*	Total
**Gender**	**Male**	919	31	950
			*(3*.*3% [2*.*2%–4*.*6%])*	
	**Female**	1078	21	1099
			*(1*.*9% [1*.*2%–2*.*9%])*	
	**Total**	**1997**	**52**	**2049**
			***(2*.*5% [1*.*9%–3*.*3%])***	
**Age group**	**5–14 years**	822	34	856
			*(4*.*0% [2*.*8%–5*.*5%])*	
	**15–24 years**	363	8	371
			*(2*.*2% [0*.*9%–4*.*2%])*	
	**25 years +**	812	10	822
			*(1*.*2% [0*.*6%–2*.*2%])*	
	**Total**	**1997**	**52**	**2049**
			***(2*.*5% [1*.*9%–3*.*3%])***	

### Cattle seropositivity

Serological testing was carried out for 955 cattle, of which 100 had serological evidence of *C*. *burnetii* exposure, giving an overall (raw) cattle seroprevalence of 10.5% (95% CI = 8.6%–12.6%). Seroprevalence was higher in female cattle (11.2%), compared to males (9.1%), although this was not statistically significant (Pearson's Chi-squared test *p* = 0.37, see Table 3).

**Table 3:** Cattle serological results, by gender (note, gender was not recorded for 3 observations).

	Seronegative	Seropositive *(seroprevalence [95% CI])*	Total
**Male**	299	30	329
		*(9*.*1% [6*.*2%–12*.*8%])*	
**Female**	553	70	623
		*(11*.*2% [8*.*9%–14*.*0%])*	
**Unknown**	3	0	**3**
		*(0% [0%–80*.*6%])*	
**Total**	**855**	**100**	**955**
		***(10*.*5% [8*.*6%–12*.*6%])***	

### Spatial analysis

**Fig 2:**
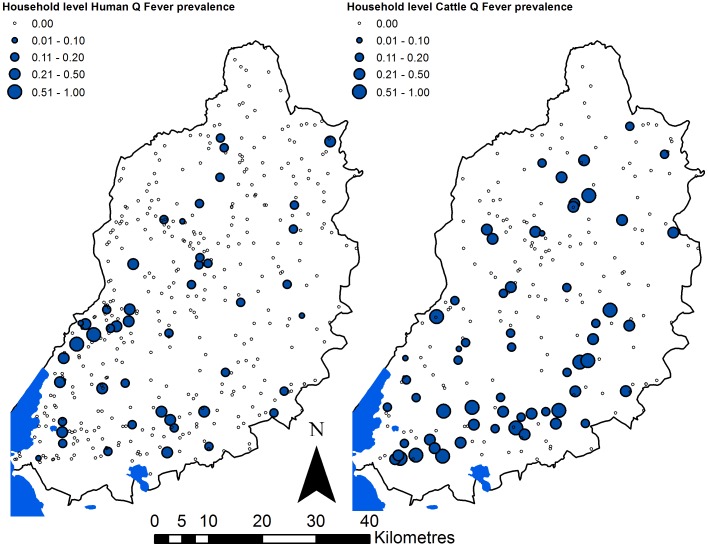
Proportion of household inhabitants seropositive for Q fever for humans (left panel) and cattle (right panel). Note the sample sizes for each individual point are small, as they are the number of residents or cattle per household.

**Fig 3:**
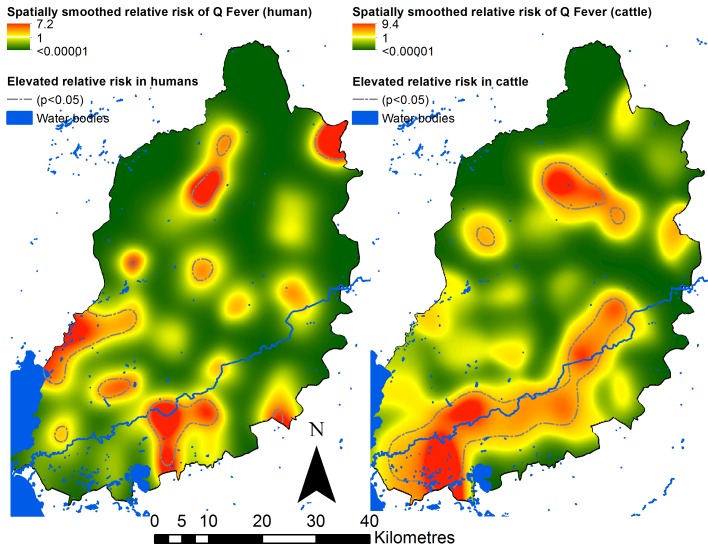
Spatially smoothed relative risks of Q fever seropositivity in humans (left panel) and cattle (right panel).

The household level human and cattle results are illustrated spatially in Fig 2, and spatially smoothed relative risks are illustrated in Fig 3. These figures indicate several areas with significantly elevated relative risk of exposure in both human and cattle populations, although the areas of elevated relative risk in humans do not clearly correspond to the areas of elevated relative risk in cattle. The main area of elevated relative risk in cattle corresponded to a major river (the Nzoia) flowing from the east of the study area, into Lake Victoria in the south west (see Fig 3).

In cattle-keeping households, household level correlation between human and cattle seroprevalence was 0.02, indicating that human exposure to Q fever is not correlated with cattle exposure within the same household. Cross–correlogram analysis did not indicate any obvious spatial cross-correlation between human and livestock exposure (see S1 Fig). Univariable logistic regression analysis results are presented in S1 Table and S2 Table, for humans and cattle respectively.

### Human regression results

The final multivariable model for human seropositivity included four individual level covariates (see Table 4). Those in the age groups 15 to 24 and 25 and over had decreased odds of exposure when compared to those aged five to 14 (odds ratio [OR] = 0.48, *p* = 0.08 [15 to 24] and OR = 0.19, *p* < 0.001 [25 and over]). Ethnic origin was also included in the final model, with significantly increased odds of exposure in Luo and Samia ethnic groups when compared to those of Luhya origin (OR = 2.47, *p* = 0.02 [Luo] and OR = 3.80, *p* = 0.002 [Samia]). In addition, those who were involved in grazing livestock on a less than daily basis, or never, had reduced odds of exposure when compared to those involved in grazing on a daily basis (OR = 0.28, *p* = 0.003 [less than daily] and OR = 0.34, *p* = 0.002 [never]); and those who had not dealt with animal abortion materials in the past 12 months had lower odds of exposure than those who had (OR = 0.13, *p* = 0.007). After accounting for individual level risk factors for human *C*. *burnetii* exposure, no household level covariates contributed significantly to the model fit. The AUC was 0.87, indicating a good model fit.

**Table 4:** Final multivariable mixed-effects logistic regression results for human seropositivity.

Covariate	Category	Coefficient	OR *(95% confidence interval)*	p-value
**Intercept**		-1.08		
**Age group**	**5–14**	Ref		
	**15–24**	-0.73	0.48 *(0*.*21–1*.*09)*	0.08
	**25 +**	-1.68	0.19 *(0*.*08–0*.*41)*	<0.001
**Ethnic origin**	**Luhya**	Ref		
	**Luo**	0.90	2.47 *(1*.*16–5*.*25)*	0.02
	**Samia**	1.33	3.80 *(1*.*63–8*.*86)*	0.002
	**Other**	0.007	1.01 *(0*.*35–2*.*91)*	0.99
**Frequency involved in grazing livestock**	**Daily**	Ref		
	**Less often**	-1.27	0.28 *(0*.*12–0*.*66)*	0.003
	**Never**	-1.09	0.34 *(0*.*17–0*.*66)*	0.002
**Dealt with animal abortus**	**Yes**	Ref		
	**No**	-2.01	0.13 *(0*.*03–0*.*57)*	0.007

### Cattle regression results

The final multivariable model for cattle seropositivity included the origin of the animal and precipitation (see Table 5). Cattle that had originated outside of the homestead (purchased at market or from another homestead) had increased odds of exposure when compared to those that were bred on the homestead (OR = 2.54, *p* = 0.0001). After accounting for this individual level risk factor, annual precipitation was also significantly correlated with the odds of exposure, with decreasing odds in areas with higher rainfall amounts (OR = 0.82, *p* = 0.002 [100 mm change in precipitation]). The AUC for the final multivariable cattle model was 0.86, indicating a good fit to the data.

**Table 5:** Final multivariable mixed-effects logistic regression results for cattle seropositivity.

Covariate	Category	Coefficient	OR *(95% confidence interval)*	p-value
**Origin of animal**	**Bred in homestead**	Ref		
	**Elsewhere**	0.93	2.54 *(1*.*57–4*.*11)*	0.0001
**Precipitation**	**(per 100 mm)**	-0.19	0.82 *(0*.*73–0*.*93)*	0.002

## Discussion

This research indicates a seroprevalence of *C*. *burnetii* of 2.5% in humans and 10.5% in cattle in a rural, livestock keeping area of western Kenya. Logistic regression analysis indicated that seroprevalence in humans varies by age and ethnic group, with cattle contact patterns also influencing the risk of seropositivity. Cattle that are brought onto a homestead, following purchase at a market or another homestead have a higher seroprevalence for *C*. *burnetii* than those bred on the homestead. Seroprevalence in cattle also correlates with precipitation levels. These results provide some much needed evidence regarding *C*. *burnetii* seroprevalence and risk factors for exposure in both humans and livestock, given the current lack of evidence to support the development of interventions in resource-poor settings.

The seroprevalence detected in humans and cattle (2.5% and 10.5% respectively) lie at the lower end of the range of estimates reported elsewhere in Africa, based on a recent systematic review which included evidence from 51 previous studies [5], and are substantially lower than the seroprevalences recently reported in western Kenya (28.3% in cattle and 30.9% in humans) [21]. This highlights the spatial heterogeneity of *C*. *burnetii* seroprevalence and, thus, exposure to *C*. *burnetii*, which has also been observed in other regions of the world [1,31]. The current lack of evidence regarding the influence of environmental, socio-economic and behavioural factors on environmental contamination with *C*. *burnetii*, pathogen survival in the environment and human and livestock exposure limits our ability to explain observed spatial heterogeneity in seroprevalence. The use of varying serological testing protocols and the potential presence of bias in previous studies may also contribute to these observed differences.

Examination of the spatial distribution of *C*. *burnetii* seropositivity from this study highlights heterogeneity in exposure, particularly amongst cattle: cattle exposure appears to be more common in the south of the study area, in proximity to the river Nzoia. Spatial clustering of *C*. *burnetii* has been observed elsewhere [14], and there is some evidence that proximity (or access) to water bodies may be a risk factor for both livestock and human infections [32,33]. Here, although the univariable analysis did indicate a statistically significant relationship between (cattle) exposure and distance to water bodies (S2 Table), the final multivariable model for cattle seropositivity did not include proximity to water bodies. The area which appeared to have greater pathogen exposure is also the part of the study area which experiences the lowest annual precipitation volumes: precipitation was included in the final multivariable model, with lower odds of exposure in areas with higher precipitation volumes. This finding corresponds with previous studies which suggest that dry conditions, in terms of precipitation, water table depth and soil types, are related to increased risk of *C*. *burnetii* infection in humans and animals [15,34–36]. An increased risk of exposure in drier conditions may be expected based on the transmission cycle: transmission usually occurs via inhalation of contaminated materials, such as environmental dust, which is more likely to be produced from dry soils or other materials.

In addition to precipitation, the animal origin was significantly associated with cattle seropositivity: cattle acquired from outside of the homestead had increased odds of exposure compared to those bred on the homestead. Of the 439 cattle not born on their homestead, the majority (75%) had been purchased at market. Seroprevalence was 13.3% in those purchased at market, 18.2% (n = 55) in those purchased from another homestead in the same village, and 20% (n = 45) in those purchased from another homestead in a different village, compared with 7.2% (n = 513) in those bred on the homestead. These results indicate substantially higher prior exposure to the pathogen in purchased animals, which may be related to contact with other livestock herds; importation of livestock from areas with higher prevalence of *C*. *burnetii*; high levels of environmental contamination in livestock markets; or an increased likelihood that farmers will sell animals suffering from Q fever related morbidity (e.g. those with reduced fertility). Based on this finding, markets may present an important location to implement control measures, for example based on pen-side testing and vaccination (which can reduce shedding in pre-exposed livestock [37]), with the potential to integrate testing and intervention for several livestock diseases. Contamination of livestock markets may also pose a risk for human health, due to the non-vegetated dusty ground which is generally present in these settings. Previous research has identified the movement of sheep through specific areas as a cause of human outbreaks [1,38], and a human outbreak has also resulted from a single event at a market [39]. Given the current lack of control for *C*. *burnetii* in many regions, the potential role of markets in disease spread and implications for human health should be a priority for future research, with a focus on the development of market-based interventions. Univariable analysis indicated that female cows which had not previously calved had lower odds of exposure to those which had previously calved, although this variable was not included in the final multivariable model. This supports previous evidence that nulliparous cows are less likely to be infected, even within infected herds [40], suggesting that cattle prior to first calving should be a priority for vaccination, if such intervention was made available.

Although some areas of significantly elevated relative risk were observed for human exposure, this was less pronounced than for cattle exposure. Univariable analysis indicated that only one environmental factor, proportion of land surrounding the homestead that was vegetated, was significantly correlated with human exposure (positive association, *p* = 0.05). This is converse to previous research elsewhere, which found a higher risk of human infection in areas with less vegetation cover, presumably related to the propensity for dust generation in non-vegetated areas [36]. The proportion of land that was vegetated was not included in the final multivariable model for human seropositivity: this may indicate that within this specific setting, environmental factors are less important for human exposure than for cattle exposure to *C*. *burnetii*.

The variables included in the final multivariable model for human exposure were age group, ethnic origin, frequency of involvement in livestock grazing, and recent involvement in disposal of animal abortion materials. Seroprevalence was highest in the youngest age group (5 to 14 years): this finding corresponds well with previous studies in different African countries [16,19,20], but in other (high-income) settings seroprevalence is commonly higher in older age groups [7,33,41]. The higher seroprevalence in children may be due to behavioural factors which increase exposure due to contact with contaminated soils (e.g. playing) or materials (e.g. dealing with infected livestock birthing materials) in combination with waning antibody levels in adults [42,43]. The variation in exposure to *C*. *burnetii* by ethnic origin has also been demonstrated for cattle in Cameroon: ethnic origin of cattle owners was associated with seroprevalence in cattle [17]. Analysis of various potential explanatory factors (e.g. behaviours, husbandry practices) by ethnic origin did not reveal any potential explanations. However, the geographical distributions of ethnic groups vary, which may, in part, explain the variations in seroprevalence. These findings indicate that either: (1) socio-cultural factors that vary by ethnic group alter exposure risk, or (2) spatial distributions of the different ethnic groups result in varying exposure risk, due to landscape factors. The current data do not allow for a full explanation for this, but future quantitative and qualitative work may provide further insights. The significance of frequency of involvement in livestock grazing (those involved in grazing on a daily basis had larger odds of exposure) and dealing with animal abortion materials (larger odds of exposure) are coherent with our present understanding of the transmission cycle for *C*. *burnetii*. These findings highlight the important influence of livestock contact on transmission of *C*. *burnetii*.

There was no evidence of correlation between cattle and human seroprevalence at the household level, or when accounting for spatial proximity between households. This indicates that a significant proportion of human exposure to Q fever may occur due to contact with, or environmental contamination by, livestock owned by other households. In addition, the serological protocol used does not differentiate between recent and historical exposure, so the unknown timing of exposure may contribute to this observed discordance. The spatial distribution of surveyed households was relatively sparse, which limits the ability of cross-correlation analyses to detect spatial correlation between human and cattle Q fever results (i.e. we do not have data from immediate neighbours): similar analysis using data from a higher resolution study (e.g. surveying every household within a small study area) could be used to provide further evidence with regards to patterns of cattle to human transmission.

Consumption of raw milk has previously been associated with risk of Q fever infection [44]: in this study population we did not find any evidence of a correlation between consumption of milk and seropositivity. Less than 1% of individuals reported consumption of raw milk, and 16.8% of individuals reported consumption of soured milk (which may be made from raw, unpasteurised milk), with the majority of individuals reporting a combination of preparation methods, including boiling.

Interpretation of these results should bear in mind diagnostic limitations. This study made use of enzyme linked immunosorbent assay (ELISA) methods, although the immunofluorescent assay test (IFAT) is generally regarded as the gold standard method for serological detection of Q fever infection [43]. However, the application of IFAT is labour intensive, making it less suitable for use in large scale surveys [45–47]. Previous evidence indicates that ELISA has generally high sensitivity and specificity in cattle (90–95% and 97–100% respectively) [48], with slightly lower sensitivity and specificity in humans (80–95% and 63%–99% respectively) [43,47–49]. This study has focussed on evidence of Q fever exposure in humans and cattle. However, small ruminants such as sheep and goats are also susceptible: further studies in this area should also incorporate small ruminants to provide a more comprehensive picture of the epidemiology of this disease. Our results did not demonstrate a significant relationship between ownership of sheep or goats and seropositivity in either humans or cattle.

In conclusion, the evidence presented here supports previous demonstrations of spatial heterogeneity in *C*. *burnetii* seroprevalence in both humans and livestock. These findings suggest that environmental factors may influence directly the spatial patterns of exposure in livestock populations, although livestock trade and cultural husbandry patterns have also been implicated. Environmental factors appear to be less important in human exposure patterns, while socio-demographics and behaviours related to livestock contact appear to be the most important factors. The role of livestock markets in *C*. *burnetii* transmission should be further investigated: livestock markets may provide suitable targets for the development of interventions in the future.

## Supporting Information

S1 Checklist STROBE checklist. (DOC)

S1 Appendix Serological testing methods, further details. (DOCX)

S1 Fig Cross-correlogram illustrating correlation between human and cattle seroprevalence at increasing distances. (TIFF)

S1 Table Univariable results for human seropositivity from mixed-effects logistic regression analysis. (DOCX)

S2 Table Univariable results for cattle seropositivity from mixed-effects logistic regression analysis. (DOCX)

## References

[1] Georgiev M, Afonso A, Neubauer H, Needham H, Thiéry R, Rodolakis A, et al Q fever in humans and farm animals in four European countries, 1982 to 2010. Eurosurveillance. 2013;18: 1–13. 23449232

[2] Halliday JEB, Allan KJ, Ekwem D, Cleaveland S, Kazwala RR, Crump JA. Endemic zoonoses in the tropics: a public health problem hiding in plain sight. Veterinary Record. 2015;176: 220–225. 10.1136/vr.h798 25722334 PMC4350138

[3] Hilbink F, Penrose M, Kovacova E, Kazar J. Q fever is absent from New Zealand. International Journal of Epidemiology. 1993;22: 945–949. 10.1093/ije/22.5.945 8282477

[4] Cutler SJ, Bouzid M, Cutler RR. Q fever. J Infect. 2007;54: 313–318. 10.1016/j.jinf.2006.10.048 17147957

[5] Vanderburg S, Rubach MP, Halliday JEB, Cleaveland S, Reddy EA, Crump JA. Epidemiology of Coxiella burnetii infection in Africa: a OneHealth systematic review. PLoS Negl Trop Dis. 2014;8: e2787 10.1371/journal.pntd.0002787 24722554 PMC3983093

[6] Agerholm JS. Coxiella burnetii associated reproductive disorders in domestic animals-a critical review. Acta Veterinaria Scandinavica. 2013;55: 13 10.1186/1751-0147-55-13 23419216 PMC3577508

[7] Raoult D, Marrie T, Mege J. Natural history and pathophysiology of Q fever. The Lancet Infectious Diseases. 2005;5: 219–226. 10.1016/S1473-3099(05)70052-9 15792739

[8] Woldehiwet Z. Q fever (coxiellosis): epidemiology and pathogenesis. Research in Veterinary Science. 2004;77: 93–100. 10.1016/j.rvsc.2003.09.001 15196898

[9] Ayres JG, Flint N, Smith EG, Tunnicliffe WS, Fletcher TJ, Hammond K, et al Post-infection fatigue syndrome following Q fever. QJM. 1998;91: 105–123. 10.1093/qjmed/91.2.105 9578893

[10] ECDC. Risk assessment on Q fever Stockholm: European Centre for Disease Prevention and Control; 2010.

[11] Raoult D. Chronic Q fever: Expert opinion versus literature analysis and consensus. Journal of Infection. 2012;65: 102–108. 10.1016/j.jinf.2012.04.006 22537659

[12] Maurin M, Raoult D. Q fever. Clinical Microbiology Reviews. 1999;12: 18–53. 10.1128/cmr.12.4.518 PMC88923 10515901

[13] Kersh GJ, Wolfe TM, Fitzpatrick KA, Candee AJ, Oliver LD, Patterson NE, et al Presence of Coxiella burnetii DNA in the Environment of the United States, 2006 to 2008. Appl Environ Microbiol. 2010;76: 4469–4475. 10.1128/AEM.00042-10 20472727 PMC2897457

[14] Commandeur M, Jeurissen L, van der Hoek W, Roest H-J, Hermans T (C. ML. Spatial relationships in the Q fever outbreaks 2007–2010 in the Netherlands. International Journal of Environmental Health Research. 2014;24: 137–157. 10.1080/09603123.2013.800963 23802588

[15] Tissot-Dupont H, Amadei M-A, Nezri M, Raoult D. Wind in November, Q Fever in December. Emerg Infect Dis. 2004;10: 1264–1269. 10.3201/eid1007.030724 15324547 PMC3323349

[16] van der Hoek W, Sarge-Njie R, Herremans T, Chisnall T, Okebe J, Oriero E, et al Short Communication: Prevalence of antibodies against Coxiella burnetii (Q fever) in children in The Gambia, West Africa. Trop Med Int Health. 2013;18: 850–853. 10.1111/tmi.12116 23600611

[17] Mazeri S, Scolamacchia F, Handel IG, Morgan KL, Tanya VN, Bronsvoort BM deC. Risk factor analysis for antibodies to Brucella, Leptospira and C. burnetii among cattle in the Adamawa Region of Cameroon: a cross-sectional study. Trop Anim Health Prod. 2012;45: 617–623. 10.1007/s11250-012-0268-0 23117621

[18] Scolamacchia F, Handel IG, Fèvre EM, Morgan KL, Tanya VN, de C. Bronsvoort BM. Serological Patterns of Brucellosis, Leptospirosis and Q Fever in Bos indicus Cattle in Cameroon. PLoS ONE. 2010;5: e8623 10.1371/journal.pone.0008623 20098670 PMC2809085

[19] Kobbe R, Kramme S, Kreuels B, Adjei S, Kreuzberg C, Panning M, et al Q Fever in Young Children, Ghana. Emerg Infect Dis. 2008;14: 344–346. 10.3201/eid1402.070971 18258140 PMC2630046

[20] Schelling E, Diguimbaye C, Daoud S, Nicolet J, Boerlin P, Tanner M, et al Brucellosis and Q-fever seroprevalences of nomadic pastoralists and their livestock in Chad. Preventive Veterinary Medicine. 2003;61: 279–293. 10.1016/j.prevetmed.2003.08.004 14623412

[21] Knobel DL, Maina AN, Cutler SJ, Ogola E, Feikin DR, Junghae M, et al Coxiella burnetii in humans, domestic ruminants, and ticks in rural western Kenya. American Journal of Tropical Medicine and Hygiene. 2013;88: 513–518. 10.4269/ajtmh.12-0169 23382156 PMC3592534

[22] Maina AN, Farris CM, Odhiambo A, Jiang J, Laktabai J, Armstrong J, et al Q Fever, Scrub Typhus, and Rickettsial Diseases in Children, Kenya, 2011–2012. Emerging Infectious Diseases. 2016;22: 883–886. 10.3201/eid2205.150953 27088502 PMC4861507

[23] DePuy W, Benka V, Massey A, Deem SL, Kinnaird M, O’Brien T, et al Q fever risk across a dynamic, heterogeneous landscape in Laikipia County, Kenya. Ecohealth. 2014;11: 429–433. 10.1007/s10393-014-0924-0 24604546

[24] Njeru J, Henning K, Pletz MW, Heller R, Neubauer H. Q fever is an old and neglected zoonotic disease in Kenya: a systematic review. BMC Public Health. 2016;16 10.1186/s12889-016-2929-9 27048480 PMC4822290

[25] Hijmans RJ, Cameron SE, Parra JL, Jones PG, Jarvis A. Very high resolution interpolated climate surfaces for global land areas. Int J Climatol. 2005;25: 1965–1978. 10.1002/joc.1276

[26] Wardrop NA. Landcover classification: western Kenya, 2010. [Internet]. University of Southampton; 2015. Report No.: 10.5258/SOTON/383135 Available: http://eprints.soton.ac.uk/383135/

[27] Jarvis A, Reuter H, Nelson A, Guevara E. Hole-filled seamless SRTM data V4 [Internet]. International Centre for Tropical Agriculture (CIAT); 2008 Available: http://srtm.csi.cgiar.org

[28] WorldPop. Worldpop alpha version 2010 estimates of numbers of people per grid square [Internet]. 2013. Available: http://www.worldpop.org.uk/data/summary/?contselect=Africa&countselect=Kenya&typeselect=Population2010

[29] Wardrop NA, Thomas LF, Atkinson PM, de Glanville WA, Cook EA, Wamae C, et al The Influence of Socio-economic, Behavioural and Environmental Factors on Taenia spp. Transmission in Western Kenya: Evidence from a Cross-sectional Survey in Humans and Pigs. PLoS Neglected Tropical Diseases. 2015;9: e0004223 10.1371/journal.pntd.0004223 26641459 PMC4671581

[30] Davies T, Hazelton M, Marshall J. Sparr: analyzing spatial relative risk using fixed and adaptive kernel density estimation in R. Journal of Statistical Software. 2011;39: 1–14. 10.18637/jss.v039.i01

[31] Harris P, Eales KM, Squires R, Govan B, Norton R. Acute Q fever in northern Queensland: variation in incidence related to rainfall and geographical location. Epidemiology & Infection. 2013;141: 1034–1038. 10.1017/S0950268812001495 22882795 PMC9151899

[32] Czaplicki G, Houtain J-Y, Mullender C, Porter SR, Humblet M-F, Manteca C, et al Apparent prevalence of antibodies to Coxiella burnetii (Q fever) in bulk tank milk from dairy herds in southern Belgium. The Veterinary Journal. 2012;192: 529–531. 10.1016/j.tvjl.2011.08.033 21962829

[33] Whitney EAS, Massung RF, Candee AJ, Ailes EC, Myers LM, Patterson NE, et al Seroepidemiologic and occupational risk survey for Coxiella burnetii antibodies among US veterinarians. Clin Infect Dis. 2009;48: 550–557. 10.1086/596705 19191638

[34] Nogareda C, Jubert A, Kantzoura V, Kouam MK, Feidas H, Theodoropoulos G. Geographical distribution modelling for Neospora caninum and Coxiella burnetii infections in dairy cattle farms in northeastern Spain. Epidemiology & Infection. 2013;141: 81–90. 10.1017/S0950268812000271 22370223 PMC9152041

[35] Prabhu M, Nicholson WL, Roche AJ, Kersh GJ, Fitzpatrick KA, Oliver LD, et al Q Fever, Spotted Fever Group, and Typhus Group Rickettsioses Among Hospitalized Febrile Patients in Northern Tanzania. Clin Infect Dis. 2011;53: e8–e15. 10.1093/cid/cir411 21810740 PMC3148261

[36] van der Hoek W, Hunink J, Vellema P, Droogers P. Q fever in The Netherlands: the role of local environmental conditions. International Journal of Environmental Health Research. 2011;21: 441–451. 10.1080/09603123.2011.574270 21563011

[37] Courcoul A, Hogerwerf L, Klinkenberg D, Nielen M, Vergu E, Beaudeau F. Modelling effectiveness of herd level vaccination against Q fever in dairy cattle. Veterinary Research. 2011;42: 68 10.1186/1297-9716-42-68 21605376 PMC3125226

[38] Dupuis G, Petite J, Péter O, Vouilloz M. An important outbreak of human Q fever in a Swiss Alpine valley. Int J Epidemiol. 1987;16: 282–287. 10.1093/ije/16.2.282 3301708

[39] Porten K, Rissland J, Tigges A, Broll S, Hopp W, Lunemann M, et al A super-spreading ewe infects hundreds with Q fever at a farmers’ market in Germany. BMC Infectious Diseases. 2006;6: 147 10.1186/1471-2334-6-147 17026751 PMC1618839

[40] Taurel A-F, Guatteo R, Joly A, Seegers H, Beaudeau F. Seroprevalence of Q fever in naturally infected dairy cattle herds. Preventive Veterinary Medicine. 2011;101: 51–57. 10.1016/j.prevetmed.2011.05.005 21645936

[41] Boden K, Brasche S, Straube E, Bischof W. Specific risk factors for contracting Q fever: Lessons from the outbreak Jena. International Journal of Hygiene and Environmental Health. 2014;217: 110–115. 10.1016/j.ijheh.2013.04.004 23707055

[42] Teunis PFM, Schimmer B, Notermans DW, Leenders AC a. P, Wever PC, Kretzschmar MEE, et al Time-course of antibody responses against Coxiella burnetii following acute Q fever. Epidemiol Infect. 2013;141: 62–73. 10.1017/S0950268812000404 22475210 PMC9152070

[43] Fournier P-E, Marrie TJ, Raoult D. Diagnosis of Q Fever. J Clin Microbiol. 1998;36: 1823–1834. 9650920 10.1128/jcm.36.7.1823-1834.1998 PMC104936

[44] Signs KA, Stobierski MG, Gandhi TN. Q Fever Cluster Among Raw Milk Drinkers in Michigan, 2011. Clinical Infectious Diseases. 2012;55: 1387–1389. 10.1093/cid/cis690 22893578

[45] Blaauw GJ, Notermans DW, Schimmer B, Meekelenkamp J, Reimerink JHJ, Teunis P, et al The application of an enzyme-linked immunosorbent assay or an immunofluorescent assay test leads to different estimates of seroprevalence of Coxiella burnetii in the population. Epidemiol Infect. 2012;140: 36–41. 10.1017/S0950268811000021 21320371

[46] Ohlson A, Malmsten J, Frössling J, Bölske G, Aspán A, Dalin A-M, et al Surveys on Coxiella burnetii infections in Swedish cattle, sheep, goats and moose. Acta Veterinaria Scandinavica. 2014;56: 39 10.1186/1751-0147-56-39 25007979 PMC4112654

[47] Meekelenkamp JCE, Schneeberger PM, Wever PC, Leenders ACAP. Comparison of ELISA and indirect immunofluorescent antibody assay detecting Coxiella burnetii IgM phase II for the diagnosis of acute Q fever. European Journal of Clinical Microbiology & Infectious Diseases. 2011;31: 1267–1270. 10.1007/s10096-011-1438-0 21997772

[48] Dean AS, Bonfoh B, Kulo AE, Boukaya GA, Amidou M, Hattendorf J, et al Epidemiology of Brucellosis and Q Fever in Linked Human and Animal Populations in Northern Togo. Samuel JE, editor. PLoS ONE. 2013;8: e71501 10.1371/journal.pone.0071501 23951177 PMC3741174

[49] Péter O, Dupuis G, Bee D, Lüthy R, Nicolet J, Burgdorfer W. Enzyme-linked immunosorbent assay for diagnosis of chronic Q fever. J Clin Microbiol. 1988;26: 1978–1982. 3053757 10.1128/jcm.26.10.1978-1982.1988 PMC266801

