# Supplementary material for: The Sero-epidemiology of Coxiella burnetii in Humans and Cattle, Western Kenya: Evidence from a Cross-Sectional Study
Source: PLoS Negl Trop Dis. 2016 Oct 7;10(10):e0005032. doi: 10.1371/journal.pntd.0005032 (PMC5055308; doi:10.1371/journal.pntd.0005032)
Supplement: S1 Appendix — (DOCX) [file pntd.0005032.s002.docx]

**Serological testing methods.**

The Serion ELISA Classic *Coxiella burnetti* Phase 2 IgG kit (Virion/Serion, Würzburg, Germany) was used to screen the human sera for antibodies to Q fever, which indicate past infection, using manufacturer’s instructions (1–3). Microtest plates were provided with *C. burnetti* antigen. Samples were diluted 1:500 in diluent and 100μL of diluted samples and control sera were pipetted into wells. Plates were incubated at 37°C for 60 minutes in a moist chamber then washed 4 times with buffer. 100μL of conjugate was added to wells and incubated at 37°C for 30 minutes in a moist chamber and then plates were washed 4 times. 100μL of substrate was added to wells and plates incubated at 37°C for 30 minutes in a moist chamber. Finally 100μL of stop solution was added to wells and plates read at 405nm and 630nm. A correction factor, which was calculated by dividing the reference optical density (OD) of the standard serum with the current OD of the standard serum, was used to account for inter-assay variability. All measured values of samples were multiplied by the correction factor and subsequently used to assign samples as seropositive or seronegative, according to manufacturer recommendations.

Cattle serological testing was performed at the International Livestock Research Institute (ILRI) Nairobi laboratory using the CHEKIT Q Fever Antibody ELISA Test Kit (IDEXX Laboratories, Wetherby, UK). This assay detects antibodies to *C. burnetti* in ruminant serum, plasma and milk samples. Sera were prediluted 1:400 using CHECKIT wash solution. 100μl of diluted samples and controls were dispensed into wells of a precoated microtitre plate and incubated at 37ºC for 60 minutes. The plate was washed with approximately 300μl CHEKIT wash solution 3 times. 100μl of conjugate was added to each well and incubated at 37ºC for 60 minutes in a humid chamber. The plate was washed with approximately 300μl CHEKIT wash solution 3 times. 100μl of TMB substrate was added to each well and the plate incubated at room temperature for 15 minutes. 100μl of stop solution was added to each well and the results read at a wavelength of 450nm. The OD results of duplicate samples were averaged and the following equation applied to the results:

𝑉𝑎𝑙𝑢𝑒 % = 𝑂𝐷sample – 𝑂𝐷neg / 𝑂𝐷pos – 𝑂𝐷neg 𝑥 100%

The sero-status of each sample was then determined according to manufacturer recommendations.

**References**

1. Blaauw GJ, Notermans DW, Schimmer B, et al. The application of an enzyme-linked immunosorbent assay or an immunofluorescent assay test leads to different estimates of seroprevalence of Coxiella burnetii in the population. Epidemiol Infect. **2012**; 140(1):36–41.

2. Péter O, Dupuis G, Bee D, Lüthy R, Nicolet J, Burgdorfer W. Enzyme-linked immunosorbent assay for diagnosis of chronic Q fever. J Clin Microbiol. **1988**; 26(10):1978–1982.

3. Waag D, Chulay J, Marrie T, England M, Williams J. Validation of an enzyme immunoassay for serodiagnosis of acute Q fever. Eur J Clin Microbiol Infect Dis. **1995**; 14(5):421–427.
