# Supplementary figures and images for: The Sero-epidemiology of Coxiella burnetii in Humans and Cattle, Western Kenya: Evidence from a Cross-Sectional Study
Source: PLoS Negl Trop Dis. 2016 Oct 7;10(10):e0005032. doi: 10.1371/journal.pntd.0005032 (PMC5055308; doi:10.1371/journal.pntd.0005032)

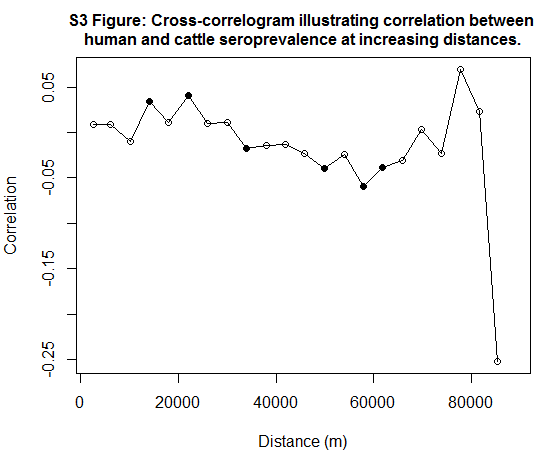

Supplement: S1 Fig — (TIFF) [file pntd.0005032.s003.tiff]
