# Supplementary material for: The Sero-epidemiology of Coxiella burnetii in Humans and Cattle, Western Kenya: Evidence from a Cross-Sectional Study
Source: PLoS Negl Trop Dis. 2016 Oct 7;10(10):e0005032. doi: 10.1371/journal.pntd.0005032 (PMC5055308; doi:10.1371/journal.pntd.0005032)
Supplement: S1 Table — (DOCX) [file pntd.0005032.s004.docx]

**S1 Table: Univariable results for human seropositivity from mixed-effects logistic regression analysis.** * Indicates variables that were statistically significant (*p* < 0.05). ** Variable was centred and scaled prior to analysis.

| **Covariate** | **Category** | **Regression**  **coefficient** | ***P*-value** |
| --- | --- | --- | --- |
| Age group | 5 – 14  15 – 24  25+ | ref  -0.60  -1.22 | 0.15  0.001* |
| Gender | Female  Male | ref  0.60 | 0.05* |
| Ethnic background | Luhya  Luo  Samia  Other | ref  0.84  1.37  -0.001 | 0.04*  0.003*  0.998 |
| Educational attainment | None  Primary  Higher | ref  -0.14  -1.44 | 0.70  0.07 |
| Occupation | Farmer  Student  Trader  Other | ref  0.56  -0.62  -1.09 | 0.11  0.43  0.30 |
| Frequency involved in grazing livestock | Daily  Less often  Never | ref  -0.95  -0.70 | 0.02*  0.04* |
| Frequency involved in feeding livestock | Daily  Less often  Never | ref  -0.85  -0.55 | 0.05*  0.10 |
| Involved in milking cattle | Yes  No | ref  0.21 | 0.62 |
| Involved in animal births | Yes  No | ref  1.41 | 0.17 |
| Involved in handling animal abortus | Yes  No | ref  -1.68 | 0.02* |
| Involved in animal slaughter | Yes  No | ref  0.18 | 0.67 |
| Involved in dealing with animal manure | Yes  No | ref  -0.08 | 0.79 |
| Involved in animal skinning | Yes  No | ref  0.61 | 0.56 |
| Involved in burying dead animals | Yes  No | ref  0.88 | 0.40 |
| Animals present in building used for sleeping | No  Yes | ref  0.81 | 0.10 |
| Drink cow’s milk | No  Yes | ref  -0.70 | 0.17 |
| Drink goat’s milk | No  Yes | ref  -0.12 | 0.89 |
| Keep cattle | No  Yes | ref  0.38 | 0.28 |
| Keep sheep | No  Yes | ref  0.34 | 0.41 |
| Keep goats | No  Yes | ref  0.33 | 0.35 |
| History of abortion in herd | Yes  No  NA | ref  -0.68  -0.91 | 0.18  0.11 |
| Ownership of at least one seropositive cow | Yes  No  NA | ref  -0.24  -0.60 | 0.59  0.20 |
| Distance to water** |  | -0.06 | 0.72 |
| Distance to flooding land** |  | 0.03 | 0.83 |
| % land agricultural and grassland |  | 2.86 | 0.09 |
| % land flooding |  | 0.16 | 0.82 |
| % land flooding agricultural and grassland |  | 0.46 | 0.69 |
| % land swamp |  | 0.29 | 0.90 |
| % land woodland and shrubs |  | -0.24 | 0.89 |
| % land vegetated |  | 6.33 | 0.05* |
| % land water body |  | -5.88 | 0.51 |
| Mean temperature (°C x 10) |  | 0.15 | 0.39 |
| Annual precipitation (mm) |  | -0.13 | 0.44 |
| Elevation (m) |  | -0.14 | 0.42 |
| Population density (persons per hectare) |  | -0.11 | 0.6 |
