# Supplementary material for: The Sero-epidemiology of Coxiella burnetii in Humans and Cattle, Western Kenya: Evidence from a Cross-Sectional Study
Source: PLoS Negl Trop Dis. 2016 Oct 7;10(10):e0005032. doi: 10.1371/journal.pntd.0005032 (PMC5055308; doi:10.1371/journal.pntd.0005032)
Supplement: S2 Table — (DOCX) [file pntd.0005032.s005.docx]

**S2 Table: Univariable results for cattle seropositivity from mixed-effects logistic regression analysis.** * Indicates variables that were statistically significant (*p* < 0.05). ** Variable was centred and scaled prior to analysis.

| **Covariate** | **Category** | **Regression coefficient** | ***P* -value** |
| --- | --- | --- | --- |
| Number of human inhabitants | <6  6 – 10  >10 | ref  0.29  0.53 | 0.41  0.20 |
| Number of cattle | <6  6 – 10  >10 | ref  0.21  0.57 | 0.54  0.17 |
| Sheep | No  Yes | ref  0.07 | 0.84 |
| Goats | No  Yes | ref  -0.03 | 0.92 |
| History of abortion in herd | Yes  No | ref  0.08 | 0.81 |
| Cattle herded with sheep and goats | Yes  No | ref  -0.3 | 0.33 |
| Herding practice (dry season) | Single herd  Multiple herds  Tethered  Other | ref  0.29  -0.25  0.95 | 0.6  0.6  0.25 |
| Herding practice (wet season) | Single herd  Multiple herds  Tethered  Other | ref  0.47  -0.15  1.43 | 0.42  0.75  0.13 |
| Breed | Zebu  Shorthorn-Zebu  Shorthorn  Other | ref  -0.2  -0.21  -0.47 | 0.5  0.6  0.4 |
| Origin of animal | Bred in homestead  Purchased | ref  0.91 | 0.0002* |
| Gender | Female  Male | ref  -0.27 | 0.29 |
| Previously calved | Yes  No  NA (male)  Unknown | ref  -0.83  -0.67  -0.25 | 0.004*  0.02*  0.83 |
| Provides milk for household | Yes  No  Unknown  NA (male) | ref  -0.53  -0.18  -0.62 | 0.07  0.88  0.05* |
| History of abortion |  | Too few observations |  |
| At least one seropositive human in household | No  Yes | ref  0.32 | 0.39 |
| Distance to water** |  | -0.41 | 0.007* |
| Inverse distance to water** |  | 0.35 | 0.003* |
| Distance to flooding land** |  | -0.33 | 0.044* |
| % land agricultural and grassland |  | 0.34 | 0.83 |
| % land flooding |  | 1.11 | 0.05* |
| % land flooding agricultural and grassland |  | 1.60 | 0.08 |
| % land swamp |  | 0.69 | 0.74 |
| % land woodland and shrubs |  | -0.0009 | 1 |
| % land vegetated |  | 6.85 | 0.03* |
| % land water body |  | 13.0 | 0.23 |
| Mean temperature** |  | 0.39 | 0.01* |
| Precipitation** |  | -0.44 | 0.002* |
| Elevation** |  | -0.38 | 0.02* |
| Population density (persons per hectare) |  | 0.007 | 0.67 |
